# Cognitive functioning in non-clinical burnout: Using cognitive tasks to disentangle the relationship in a three-wave longitudinal study

**DOI:** 10.3389/fpsyt.2022.978566

**Published:** 2022-08-17

**Authors:** Panagiota Koutsimani, Anthony Montgomery

**Affiliations:** ^1^Department of Educational and Social Policy, School of Social Sciences, Humanities and Arts, University of Macedonia, Thessaloniki, Greece; ^2^Department of Psychology, Northumbria University Newcastle, Newcastle upon Tyne, United Kingdom

**Keywords:** burnout, cognitive functioning, cognitive impairment, depression, anxiety, perceived family support, longitudinal study

## Abstract

Burnout is often characterized by cognitive deficits and it has been associated with depression and anxiety. However, it is not clear whether cognitive impairment is a burnout consequence or employees with poor cognitive skills are more prone in developing burnout. Moreover, the exact nature of the association between burnout and depression, and burnout and anxiety is still unknown. Depression and anxiety are also related to cognitive impairments but their prospective associations are not fully understood. The aim of the present three-wave longitudinal study was to investigate the causality between cognitive functioning, burnout, depression, and anxiety among non-clinical burnout employees. The cause-effect associations of burnout with depression and anxiety were also explored. Perceived family support as a protective factor against cognitive decline, burnout, depression and anxiety was examined as well. A wide range of cognitive tasks tapping different cognitive domains were administered to employees of the general working population. Burnout, depression, anxiety, and perceived family support were assessed with self-reported questionnaires. Present results suggest that visuospatial functioning deficits are a burnout consequence and they indicate the role of automatic processing skills and executive functions in burnout onset. Additionally, current findings support that burnout is differentiated from depression and anxiety but it is reciprocally associated with the two psychological phenomena. Lastly, current results support the inclusion of perceived family support as an intervention to help individuals who suffer from mental health and cognitive difficulties.

## Introduction

Occupational chronic stress is a growing health risk factor among working populations (1) and burnout is one of its consequences. The concept of burnout is not unitary and its definition depends upon the tool used for its measurement (2). The present paper adopts the approach to burnout as provided by Maslach et al. (3). That is, burnout consists of three dimensions; exhaustion, cynicism, and reduced personal efficacy (3). Exhaustion refers to the depletion of emotional resources; cynicism concerns employees’ distancing from their work, clients etc.; reduced personal efficacy is the negative appraisal of an individual’s work resulting in low work self-esteem (4). Hereafter, the term “burnout” reflects the abovementioned three dimensions. Cognitive performance has become an important aspect in the burnout research during the past decades. Desart et al. (5) proposed a new burnout definition by including two new core symptoms; cognitive and emotional loss of control; a notion that has been supported by other scholars as well (6). Nevertheless, whether cognitive impairment is a burnout consequence or employees with lower cognitive skills are more prone to such stress effects and thus, more vulnerable to burnout is still unclear. An additional limitation concerns the lack of studies investigating non-clinical burnout, as most researchers focus on clinical populations. By taking into consideration the importance of detecting and addressing early the burnout effects, the present study focuses on the examination of non-clinical burnout employees; i.e., employees who have not received a clinical diagnosis (in contrast to the clinical burnout individuals) but report high burnout levels as measured by self-reported questionnaires and they continue working.

Burnout has also been associated with depression and anxiety as burned-out employees often suffer from these two mental health problems (7). Burnout’s comorbidity with depression and anxiety is so common that its empirical distinction from these two mental disorders has been a matter of debate among researchers (8, 9). Additionally, both depression and anxiety have been linked with cognitive impairment (10, 11). Hence, when examining the association between burnout and cognitive functioning researchers also need to consider for possible comorbidity with depression and anxiety as this association might be biased by the presence of these two mental health problems. Another research question that has not received much attention concerns whether individuals with weaker cognitive skills are more susceptible in experiencing psychological distress as a result of the everyday difficulties these individuals might experience.

### Burnout and cognitive functioning

Several studies (12, 13) and reviews (14, 15) have indicated the negative associations between burnout and cognitive functioning, with executive functioning deficits being the most prominent ones (14). Executive functions are high order cognitive functions and they include cognitive skills such as inhibition, switching (or cognitive flexibility), planning (16), reasoning, and problem solving (17). Employees with poorer executive function skills might be less able to stay focused on tasks, make decisions, and struggle with time management. All these difficulties can potentially lead to burnout onset. At the same time, as burnout levels rise they can further affect executive functions (18); a process that leads to a vicious circle. Although executive deficits are common amongst burned-out employees, they are also the most studied ones (14); overlooking potential associations between burnout and other cognitive functions.

The basic burnout conceptualization regards burnout as a three-dimensional concept. Nevertheless, the consistent associations of burnout with cognitive deficits have led several scholars (5, 6) to enhance our approach to the burnout model by arguing that cognitive deficits should also be included in the burnout definition. Schaufeli et al. (6) identified cognitive deficits as a core burnout component while the authors argued that cognitive impairment is a burnout consequence that emerges due to the lack of energy which is prominent in burnout. However, the causality between burnout and cognitive functioning should first be elucidated before cognitive impairment is included as a core burnout dimension.

Although individuals with poorer executive functioning abilities are more vulnerable to the stress effects (19), the investigation of whether cognitive impairment is a burnout consequence or employees with lower cognitive abilities are more prone in developing burnout is an under-explored research area. Most relevant studies are of a cross-sectional design while longitudinal studies mainly focus to the long-lasting effects of burnout (20, 21). To the authors’ knowledge, so far, only two studies have examined for reversed effects and they provide mixed results. Specifically, in their study, Feuerhahn et al. (22) found that executive functioning deficits were a consequence of the exhaustion burnout dimension while Lemonaki et al. (23) showed that lower cognitive flexibility abilities—but not working memory and inhibition—predicted higher burnout levels. Nevertheless, both studies focused only on the examination of executive functions while they measured this relationship only at two different time points. Hence, it still remains impossible for the form of change of these two variables over time to be determined and the results could possibly reflect measurement error and not a true change over time (24).

Although the cognitive deficits in clinical burnout have been widely recognized (21, 25), the results among non-clinical burnout populations remain inconclusive. Physiological studies suggest the negative effects of burnout as it leads to a decrease of the brain-derived neurotrophic factor (BDNF) (26); a protein which plays in important role on neuroplasticity (27). Decrease BDNF levels have been observed to mediate the negative effects of non-clinical burnout on cognitive functioning (28); a result which indicates that even at its early stages, burnout can disrupt normal brain and cognitive functioning. Studies have observed working memory deficits among non-clinical burnout employees (29) while others have failed to find cognitive impairments (30). Interestingly, some studies observed greater verbal working memory skills (31). A more recent study provides mixed results as the researchers showed that cynicism was negatively related to visuospatial skills but positively related to automatic processing skills (32); possibly indicating the employees’ activation of strategies in order to amplify their efforts during the initial burnout stages.

The sparse evidence regarding the cognitive patterns that characterize non-clinical burnout do not allow for drawing safe conclusions. Two theories that could explain the positive associations between non-clinical burnout and cognitive functioning, and in which the current study draws on, are the cognitive reserve (CR) and the self-regulation theories. The CR theory (33) suggests that when individuals carry out challenging tasks they employ the cognitive reserve process which permits them to use those cognitive strategies and brain networks that are more efficient and thus, enable them to cope with failure. Self-regulation theory (34) concerns one’s ability to inhibit a prepotent response and guide their cognitive, affective and behavioral sources for attaining a goal. That is, an individual’s self-regulation processes enable them to direct their cognitive resources on completing a task (35). Hence, non-clinical burnout employees might be still able to self-regulate and initiate the cognitive reserve in order to achieve optimal performance; a process which could possibly explain the observed greater cognitive performance in certain non-clinical burnout employees. In line with the above theoretical arguments, we hypothesize that:

**Hypothesis 1:** Non-clinical burnout will be positively related to cognitive functioning.

### Depression and anxiety

Depression and anxiety are two mental health problems that often coincide with burnout. This concurrence is so frequent which—along with their conceptual and behavioral similarities—have led to a debate among researchers on whether burnout is distinct—or another form—from these two psychological phenomena (36, 37). So far, the research evidence is inconclusive as some argue that burnout is a specific form of depression (36) while others suggest that burnout is a distinct concept (38); indicating also the importance of developing targeted clinical examinations of the working populations. Recent studies show that although burnout is accompanied by depressed mood and distressed feelings (6, 39), these feelings do not constitute robust and stable burnout characteristics, instead they might result as a reaction to burnout’s onset (39).

Regarding the relationship between burnout and depression, four theoretical models have been proposed: (1) the stability model which proposes that, although burnout and depression are associated with each other, they are independent from one another (40); (2) the burnout-as-antecedent model which suggests that burnout leads to depression and not the opposite (41); (3) the burnout-as-consequence model which assumes that depression leads to burnout and not vice versa (42); (4) the reciprocal effects model which posits that the second and third models are both true (43). Considering the relatively stable significant associations between burnout and depression when examining their predictive relationship, the most comprehensive model to consider is the reciprocal effects model. Although these models concern the burnout-depression relationship, in view of the lack of a similar theoretical framework in this study the same argumentation was assumed for the burnout-anxiety relationship.

Drawing on the above, we suggest that burnout is bidirectionally associated with depression and anxiety but it is a distinct concept. On this basis, the following hypothesis was formulated:

**Hypothesis 2:** Burnout will be moderately and positively related to depression and anxiety.

Depression and anxiety have been linked with cognitive deficits pertaining to memory (44) and executive functions (45, 46). However, most relevant studies focus on clinical populations. Ganguli et al. (47) showed that depressive symptoms among non-clinical populations were cross-sectionally, but not prospectively, associated with cognitive impairment. To the authors’ knowledge, the examination of everyday anxiety in non-clinical populations so far has received no attention as most studies focus on the effects of specific anxiety disorders on cognitive functions, such as obsessive compulsive (48) and post-traumatic disorder (49). An additional limitation concerns the insufficient evidence examining the role of cognitive functions in the onset of depression and anxiety. Studies have shown that individuals with lower cognitive abilities are more susceptible in developing depression (50) and anxiety (51). This study sought to elucidate the nature of the causality between cognitive functioning and depression, and anxiety.

Based on the above empirical argumentation we propose that:

**Hypothesis 3:** Non-clinical depression and non-clinical anxiety will be related to lower cognitive performance.

**Hypothesis 4:** Individuals with poorer cognitive abilities will exhibit higher depression and anxiety levels.

### Perceived family support

An important area in the burnout research is the investigation of non-work-related factors that can contribute to—or protect against—its onset. Most studies focus on the effects of perceived social support (friend, co-worker support) on mental health (52, 53). Considering the COVID-19 pandemic challenges and the fact that many employees had to work from home, the examination of the effects of family on employees’ mental health is of importance. Although limited, greater levels of perceived family support have been found to protect against burnout onset whereas poor perceived family support appears to enhance it (54, 55). The traditional conceptualization of depression and anxiety mainly focuses on the interpersonal factors affecting the two mental health issues. Nevertheless, although greater levels of family support can reduce mental health problems the reverse could also hold true; i.e., depression and anxiety may lead to a reduction in the quality of one’s family relationships (56). According to Coyne’s interactional theory of depression, depressed individuals provoke negative interaction patterns with others leading to a rejection from them which further increases their depressive feelings, creating a vicious circle (57). Thus, family support could act both as a consequence and contributing factor of burnout, depression and anxiety suggesting mutual reinforcing relationships (58). Although family support has been observed to facilitate cognitive performance (59), the relevant research evidence is sparse.

In line with the above empirical evidence, we hypothesize:

**Hypothesis 5**: Perceived family support will be negatively related to burnout, depression and anxiety, and positively associated with cognitive performance.

**Hypothesis 6:** Higher burnout, depression and anxiety levels will predict lower feelings of perceived family support.

## Aims of the present study

As most studies mainly focus on clinical populations, in the present study we sought to examine non-clinical populations *via* a longitudinal design. Moreover, considering that executive functions are the most studied cognitive functions, we measured a broad range of cognitive functions.

The present study adds to the current literature in four ways. Firstly, by elucidating the association between non-clinical burnout and cognitive functioning and whether cognitive impairment is a burnout consequence, or employees with poorer cognitive abilities are more susceptible in developing burnout. Next, we aim to explore the causality of non-clinical depression and anxiety with cognitive functioning. Third, we endeavor to clarify the nature of the burnout-depression and burnout and anxiety relationships. Lastly, we examine the role of perceived family support in cognitive functioning, burnout, depression, and anxiety.

## Materials and methods

### Ethics

The ethical procedures according to the declaration of Helsinki when conducting research with human participants were followed. All participants agreed and signed an informed consent form for voluntary participation prior to their participation in the research at all three times of assessment.

### Procedure

Following the methodological procedures of previous longitudinal studies that examined both cross-sectionally (12, 60, 61) and longitudinally (20, 21, 62) the burnout—cognitive functioning relationship, we conducted a baseline study in order to gain an appreciation of the participants’ baseline characteristics (32) and how they developed over time. The present study concerns the longitudinal results after the completion of all three-time measurements.

Details regarding the procedures that were followed have been previously described (32). As both brief and extended time lags can decrease the chances of detecting the effects of the independent to the depended variable (63), participants were examined within an 8 and 17-month period interval after the baseline. That is, after the completion of each wave assessment, the participants who had initially attended the cognitive evaluation were first conducted to participate in the follow-up assessment. The baseline assessment was conducted between September 2018 and January 2019.

**FIGURE 1:**
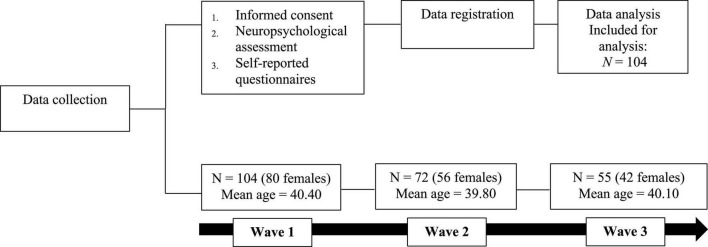
Schematic figure of the study’s procedure.

The majority of research in the area of burnout suffers from the problem of common method variance among the related survey instruments. The present research avoids this problem by involving participants in an exhaustive in-person cognitive functioning paper-and-pencil test. The structured interview lasted 60 min on average. This assessment procedure is the most reliable and valid way to assess cognitive functioning among individuals, but the interview is time-consuming and demanding for non-clinical burnout participants; and thus, it is more reliable for detecting any cognitive impairments (see Figure 1 for a schematic presentation of the procedure).

### Participants

The participants were Greek employees of the general working population. Of the 104 participants who were assessed at baseline (T1), 72 of them agreed to participate at the first follow-up (T2) and 55 at the second follow-up (T3). *Post hoc* G*Power analysis with a medium effect size (*f^2^* = 0.15) revealed a sufficient sample size to identify at least medium effects with a statistical power > 0.80 (β = 0.88) (64). The initial sample was contacted either by phone or in person; and by phone during the two follow-ups. Exclusion criteria included neurological disorders that could affect cognitive functioning (i.e., traumatic brain injury, epilepsy, multiple sclerosis).

### Questionnaires

Burnout was assessed by administering the Maslach Burnout Inventory—General Survey (MBI-GS) (65); a 16-item Likert-type scale designed to measure the three burnout subscales. All three subscales exhibited good reliabilities at all three time points with Cronbach’s alphas: α_*t*1_ = 0.90, α_*t*2_ = 0.92 and α_*t*3_ = 0.94 for exhaustion; α_*t*1_ = 0.71, α_*t*2_ = 0.79 and α_*t*3_ = 0.83 for cynicism; and α_*t*1_ = 0.84, α_*t*2_ = 0.85 and α_*t*3_ = 0.78 for personal efficacy. The McDonald’s omegas were ω_*t*1_ = 0.90, ω_*t*2_ = 0.92, ω_*t*3_ = 0.94 for exhaustion; ω_*t*1_ = 0.73, ω_*t*2_ = 0.80, ω_*t*3_ = 0.85 for cynicism; and ω_*t*1_ = 0.84, ω_*t*2_ = 0.82, ω_*t*3_ = 0.80 for personal efficacy.

The Hospital Anxiety and Depression Scale (HADS) is comprised of two 7-item subscales for measuring self-reported anxiety and depression (66) and it can be used in both patient and healthy populations (67). Scores ≥ 8 indicate potential case of depression and/or anxiety. Cronbach’s alphas were α_*t*1_ = 0.74, α_*t*2_ = 0.79 and α_*t*3_ = 0.79 for depression, and α_*t*1_ = 0.84, α_*t*2_ = 0.83 and α_*t*3_ = 0.85 for anxiety; the respective McDonald’s omegas were: ω_*t*1_ = 0.74, ω_*t*2_ = 0.79, ω_*t*3_ = 0.74 for depression ω_*t*1_ = 0.84, ω_*t*2_ = 0.84, ω_*t*3_ = 0.85 for anxiety.

The Julkunen Family Support Scale (FSS) (68) consists of 13 items and assesses individual’s subjective feelings of perceived family support; scores > 37 suggest high sense of perceived family support. Cronbach’s alphas were α_*t*1_ = 0.81, α_*t*2_ = 0.80 and α_*t*3_ = 0.82, and McDonald’s omegas were ω_*t*1_ = 0.90, ω_*t*2_ = 0.81, ω_*t*3_ = 0.80.

### Cognitive functioning

Seven different cognitive tasks were administered for assessing a wide variety of cognitive functions. Executive functions (i.e., inhibition, interference control, cognitive flexibility, switching, attention/speed of processing) were tested using the Stroop task and the Trail Making test (parts A and B) (69, 70). Auditory working memory was tested with the Digit Span task from Wechsler’s Adult Intelligence Scale Revised (WAIS-R) (71) while visuospatial working memory was assessed using the Corsi Block—Tapping Span task (backwards) (72). Visuospatial skills and visuospatial short-term and long-term memory were measured with the Taylor Complex Figure Test (73). Episodic memory was tested with the Short Story (immediate and delayed recall of a short story) (74). Prospective memory was examined with the use of a self-made test advised by Eskildsen et al. (12).

All tests were administered in a standardized sequence at all three measurements.

### Statistical analysis

All statistical analyses were performed on SPSS (v.21). One-way ANOVA compare means were conducted to explore for any potential differences between the participants who continued with the follow-ups and the participants who dropped out. Linear mixed model analysis was performed in order to examine for potential predictive relationships. One advantage of this type of analysis is that it uses the full dataset to estimate the examined associations reducing the *post hoc* volunteer bias (75). The random effects model was chosen. Model selection was based in an unstructured covariance type. The random effects model was chosen for two reasons: (1) we wanted to examine the effects of the predictor variable to the dependent across the three time points and (2) due to the importance of allowing the intercepts for each participant to be different in longitudinal studies (76). Specifically, we specified both participants and time to be correlated with random effects with a scaled identity repeated covariance type. Next, the three MBI-GS subscales were set as covariates and each cognitive task, the total scores on the HADS (depression and anxiety separately) and FSS were considered as dependent variables. Additionally, the total HADS and FSS scores (independently) were set as covariates and each cognitive task and each of the three MBI-GS subscales were set as dependent variables. As not all participants were living with someone else, the linear mixed models, where the FSS total score had been set as the predictive factor, were based on a smaller sample size (*N* = 80). Statistically significant results were examined for reversed associations. *P*-values < 0.05 were considered as statistically significant.

## Results

### Descriptive characteristics

All variables were normally distributed as skewness and kurtosis indices were within the normal range. Mahalanobis distance (77) was used in order to identify for multivariate outliers. Two multivariate outliers were detected. However, considering that exclusion of outliers in studies with multiple observations can result in a restriction bias (78), we decided to include the two outliers in the analysis.

Table 1 shows participants’ descriptive characteristics across the three time points. Although there was a statistically significant difference between groups at T1 and T3 as determined by one-way ANOVA [*F*(2, 228) = 3.482, *p* = 0.032], a Tukey *post-hoc* test did not reveal any significant differences between groups across the three time points. Thus, as the participants at all three time points were mainly missing at random, we expected unbiased estimates. Table 2 depicts the significant correlations observed between participants’ descriptive characteristics at T1 and the scores on the MBI-GS (T3) and performance on the cognitive tasks (T3). Concerning participants’ gender, male participants exhibited lower scores (*t* = -2.54, *p* < 0.05) on the personal efficacy subscale (*M* = 4.50, *SD* = 1.33) comparing to the female participants (*M* = 5.18, *SD* = 0.64). With respect to family status, as determined by one-way ANOVA there was a statistical significant difference between participants’ family status at T1 and exhaustion at T3 [*F*(2, 228) = 4.423, *p* = 0.008], and performance on the third condition on the Stroop test [*F*(2, 228) = 3.408, *p* = 0.024]. Particularly, married participants exhibited significantly lower scores on the exhaustion subscale (*M* = 2.08, *SD* = 1.43) compared to the single participants (*M* = 3.65, *SD* = 1.56). Married participants performed lower on the third Stroop condition (*M* = -0.47, *SD* = 0.78) compared to the single participants (*M* = 0.37, *SD* = 0.96). None of the participants’ descriptive characteristics were associated with the scores on the HADS (T3) and FSS (T3) (see Supplementary Material for the correlations coefficients between burnout, depression screening, anxiety, perceived family support and cognitive tasks on each measurement point).

**TABLE 1:** Demographic characteristics of the participants at T1 (*N* = 104), T2 (*N* = 72), and T3 (*N* = 55).

Characteristic	T1	T2	T3
**Age** (mean, *SD*)	40.40 (10.06)	39.80 (9.80)	40.10 (9.89)
**Years of education** (mean, *SD)*	16.82 (1.39)	16.88 (1.37)	16.90 (1.26)
**Years of working experience** (mean, *SD*)	15.20 (8.67)	15.97 (10.64)	16.18 (11.44)
	*N* (%)	*N* (%)	*N* (%)
**Children** *yes/no*	40/64 (38.5/61.5)	28/44 (38.9/61.1)	22/33 (40/60)
**Sector**			
*Public*	62 (59.6)	44 (61.1)	33 (60)
*Private*	42 (40.4)	28 (38.9)	22 (40)
**2nd occupation**	22 (21.1)	17 (23.6)	9 (20)
**Males/females**	24/80 (23.1/76.9)	16/56 (22.2/77.8)	13/42 (23.6/76.4)
**Family status**			
*Cohabitating*	8 (7.7)	8 (11.1)	7 (12.7)
*Married*	47 (45.2)	31 (43.1)	24 (43.6)
*In a relationship*	1 (1)	–	–
*Separated*	4 (3.8)	4 (5.6)	3 (5.5)
*Divorced*	3 (2.9)	–	–
*Single*	41 (39.4)	29 (40.3)	21 (38.2)
**Questionnaires**	Mean (*SD*)	Mean (*SD*)	Mean (*SD*)
Exhaustion	2.89 (1.54)	2.88 (1.56)	2.97 (1.70)
Cynicism	2.08 (1.30)	2.54 (1.45)	2.58 (1.51)
Personal efficacy	5.05 (0.96)	5.04 (0.85)	5.00 (0.88)
HADS-depression	5.02 (3.24)	5.08 (3.38)	5.69 (3.22)
HADS-anxiety	6.33 (3.82)	6.12 (3.69)	6.45 (4.00)
Family support	51.40 (8.88)	51.50 (8.82)	52.50 (8.66)

**TABLE 2:** Correlations of demographics (T1) with MBI-GS (T3) and cognitive tasks (T3) (*N* = *104).*

Variable	Exh.	CY	PE	TCFT-del.	Stroop-CW
Hours/week-main	−0.34**	0.37**	*ns*	*ns*	0.33*
Hours/week-second	ns	ns	ns	0.28*	ns
Sector		ns	ns	ns	0.29*
Years of working experience	−0.42**	ns	ns	ns	−0.36**
Family status	−0.34**	ns	ns	ns	−0.30*
No of children	−0.36**	ns	ns		
Age	−0.32*	ns	ns	ns	−0.45**
Gender	ns	ns	0.33*	ns	ns

Exh, Exhaustion; CY, Cynicism; PE, Personal Efficacy; TCFT-del., Taylor Complex Figure Test—delay condition; Stroop CW, Stroop Color-Word condition; Hours/week-main, Working hours for the main occupation; Hours/week, Working hours for the second occupation; Sector, Public and Private; Family Status, Single, In a Relationship, Cohabitating, Married, Separated, Divorced; ns, non-significant.

**p < 0.01 level (2-tailed); *p < 0.05 level (2-tailed).

## Linear mixed model analysis

### Effects of burnout, depression, anxiety and family support on cognitive performance

**FIGURE 2:**
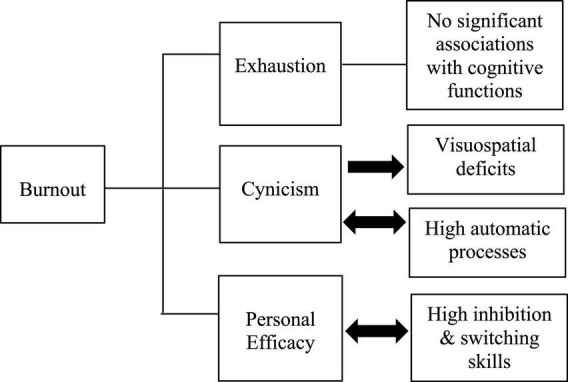
Schematic figure of the results regarding the burnout—cognitive functioning relationship.

As certain demographics were significantly correlated with the performance on certain cognitive tasks (see Table 2), we controlled for these variables. Exhaustion did not predict participants’ cognitive performance in any of the cognitive tasks. Cynicism had significant effects on cognitive performance on the TCFT-copy condition (95% CI = −0.23, −0.01, *b* = −0.12, *p* < 0.05) and on the first condition of the Stroop task (95% CI = 0.00, 0.22, *b* = 0.11, *p* < 0.05) across the three time points. When we examined for reversed associations, TCFT-copy condition did not have significant effects on cynicism [*F*(1, 5.179) = 3.60, *p* = 0.11] while performance on the first Stroop condition had significant effects on cynicism (95% CI = 0.00, 0.38, *b* = 0.19, *p* < 0.05). Personal efficacy significantly predicted participants’ performance on the incongruent Stroop condition (95% CI = 0.00, 0.02, *b* = 0.01, *p* < 0.01) (see Table 3). When examined for reversed associations, the incongruent Stroop condition also significantly predicted personal efficacy (95% CI = 0.15, 0.53, *b* = 0.28, *p* < 0.01) (see Figure 2 for a schematic presentation of the results).

**TABLE 3:** Linear mixed model analysis of MBI-GS subscales as predictive factors (*N* = 104).

	Exhaustion			Cynicism			Personal Efficacy		
Parameter	*b*	*SE b*	*95% CI*	*b*	*SE b*	*95% CI*	*b*	*SE b*	*95% CI*
Depression	0.85 +	0.14	0.57, 1.13	0.83 +	0.13	0.55, 1.11	−0.81 +	0.29	−1.42, −0.21
Anxiety	1.07 +	0.16	0.73, 1.40	0.78 +	0.16	0.44, 1.12	−1.05 +	0.28	−1.62, −0.48
Family support	ns	ns	ns	−1.17*	0.48	−2.14, −0.01	ns	ns	ns
TCFT-copy	ns	ns	ns	−0.12*	0.05	−0.23, 0.01	ns	ns	ns
Stroop-W	ns	ns	ns	0.11*	0.05	0.00, 0.22	ns	ns	ns
Stroop-CW	ns	ns	ns	ns	ns	ns	0.01 +	0.00	0.00, 0.02

*p < 0.05; +*p* < 0.01, b, unstandardized coefficient; ns, non-significant; TCFT, Taylor Complex Figure Test; Stroop-W, Stroop Word; Stroop-CW, Stroop Color Word.

### Effects of depression, anxiety on cognitive performance

No significant effects of depression screening and anxiety on cognitive performance were observed (all *ps* > 0.05).

### Effects of burnout on depression, anxiety and perceived family support

Exhaustion significantly predicted both depression screening (95% CI = 0.57, 1.13, *b* = 0.85, *p* < 0.01) and anxiety (95% CI = 0.73, 1.40, *b* = 1.07, *p* < 0.01) while it did not have any effects on perceived family support. When examined for reversed associations, both depression screening (95% CI = 0.89, 0.20, *b* = 0.149, *p* < 0.01) and anxiety (95% CI = 0.11, 0.21, *b* = 0.16, *p* < 0.01) predicted exhaustion. Cynicism was found to have significant effects on depression screening (95% CI = 0.55, 1.11, *b* = 0.83, *p* < 0.01) anxiety (95% CI = 0.44, 1.12, *b* = 0.78, *p* < 0.01) and perceived family support (95% CI = −2.14, 0.20, *b* = −1.17, *p* < 0.05). Personal efficacy had significant effects on anxiety (95% CI = −1.62, −0.48, *b* = −1.05, *p* < 0.01) and depression screening (95% CI = −1.42, −0.21, *b* = −0.81, *p* < 0.01). No significant effects of personal efficacy in perceived family support were found (see Table 4). When examined for reversed associations, depression screening (95% CI = 0.12, 0.24, *b* = 0.18, *p* < 0.01), anxiety (95% CI = 0.10, 0.20, *b* = 0.15, *p* < 0.01) and perceived family support (95% CI = −0.06, −0.07, *b* = −0.03, *p* < 0.05) had significant effects on cynicism. Moreover, both depression screening (95% CI = −0.18, −0.07, *b* = −0.12, *p* < 0.01) and anxiety (95% CI = −0.09, −0.20, *b* = −0.05, *p* < 0.01) had significant effects on personal efficacy (see Tables 4, 5).

**TABLE 4:** Linear mixed model analysis of depression and anxiety as predictive factors (*N* = 104).

	Depression			Anxiety		
Parameter	*b*	*SE b*	95% CI	*b*	*SE b*	95% CI
Exhaustion	0.14 +	0.02	0.08, 0.20	0.16 +	0.02	0.11, 0.21
Cynicism	0.18 +	0.03	0.12, 0.24	0.15 +	0.02	0.10, 0.20
Personal efficacy	−0.12 +	0.02	−0.18, −0.07	−0.05 +	0.01	−0.09, −0.02
Family support	−0.88 +	0.21	−1.30, −0.46	−0.68 +	0.19	−1.07, −0.29

**+***p* < 0.01, b, unstandardized coefficient.

**TABLE 5:** Linear mixed model analysis of perceived family support as a predictive factor (*N* = 80).

Parameter	*b*	*SE b*	95% CI
Cynicism	−0.03*	0.01	−0.06, −0.00
Depression	−0.13 +	0.03	−0.20, −0.07
Anxiety	−0.13 +	0.03	−0.20, −0.06
TCFT-delayed	0.00 +	0.00	0.00, 0.00

*p < 0.05; **+***p* < 0.01, b, unstandardized coefficient; TCFT-delayed, Taylor Complex Figure Test-delayed recall condition.

### Effects of perceived family support on depression, anxiety and cognitive performance

Perceived family support significantly predicted anxiety (95% CI = −0.20, −0.06, *b* = −0.13, *p* < 0.01) and depression screening (95% CI = −0.20, −0.07, *b* = −0.13, *p* < 0.01). When examined for reversed associations, depression screening (95% CI = −1.30, −0.46, *b* = −0.88, *p* < 0.01) and anxiety (95% CI = −1.07, −0.29, *b* = −0.68, *p* < 0.01) also had significant effects on perceived family support. With respect to cognitive functioning, perceived family support had significant effects on participants’ performance on the TCFT-delayed recall condition (95% CI = 0.00, 0.00, *b* = 0.00, *p* < 0.01). When examined for reversed associations, performance on the TCFT-delayed recall condition did not predict the levels of perceived family support [*F*(1, 163.680) = 1.90, *p* = 0.16] (see Tables 4, 5).

## Discussion

The objectives of the present study were to examine (1) the causality between non-clinical burnout and cognitive functioning, (2) the causality between self-reported depression and anxiety with cognitive performance, (3) the nature of the relationship between burnout and depression screening, and burnout and anxiety, and (4) the role of perceived family support in mental health and cognition.

### Associations between burnout and cognitive functioning

Our results did not provide robust evidence that non-clinical burnout affects cognitive performance. However, some interesting results were observed. Contrary to our expectations and previous studies (22), baseline exhaustion was not a predictive factor of cognitive functioning. Nonetheless, both cynicism and personal efficacy had significant effects on participants’ performance in certain cognitive tasks (see Figure 2). Particularly, cynicism negatively affected visuospatial abilities while it had positive effects on automatic processes. When we examined for reciprocal associations, visuospatial skills did not predict cynicism, suggesting that decreased visuospatial skills are a consequence of burnout; a cognitive deficit that has been observed in clinical burnout as well (79). By way of contrast, automatic processes had a positive and bidirectional relationship with cynicism. This observation is in line with—and somewhat can be explained by—the findings of Van Dam et al. (80). Specifically, the researchers showed that employees who reported high burnout levels were able to efficiently perform on cognitive tasks and applied high-effort strategies but they also experienced greater distress during task performance. Thus, current results could reflect employees’ struggles to apply high-effort strategies (i.e., by increasing their automatic processing skills) in order to cope with the given task. A positive and reciprocal relationship between personal efficacy and executive functions (inhibition and switching) was also observed. This result is similar to the study of Morgan et al. (81) who observed that greater personal efficacy levels were associated with greater executive functions suggesting that employees’ perception of personal efficacy might be responsible for maintaining optimal cognitive performance.

In line with the CR (33) and self-regulation (34) theories, we argued that successful self-regulation enables employees to activate their cognitive reserve processes in order to optimize their performance. Perhaps, when employees who experience high cynicism levels start to become aware of a depletion of their mental and physical energy, they start to deploy more cognitive resources such as automatic processing skills, which are effortless and implicit and thus, easier to employ. Automatic processing is based on well-learned information (e.g., word reading) and is cue-driven (i.e., is triggered by the context and it is not intentional). Hence, it is an unconscious process which requires few attentional resources and it is more likely to occur among consistent learning environments (82). Greater performance on cognitive tasks that are reliant on automatic processing, such as the Stroop task (83), could indicate that employees who are high on both cynicism and personal efficacy do not yet lack the emotional resources that are needed in order to engage in greater cognitive efforts. Self-efficacy concerns individuals’ judgments on how sufficient they are in accomplishing a task (84) and it has been found to be related with a greater sense of purpose and control (85). Perhaps, employees who are at the initial burnout stages (i.e., high cynicism levels), but also experience a high sense of self-competency, are able to successfully regulate themselves and deploy more cognitive resources in order to compensate for their difficulties.

The reverse association between automatic processes and inhibition at T1 and cynicism, and personal efficacy at T3, respectively, also emphasize the role of executive control in burnout onset. According to the strain vulnerability hypothesis (86), individuals with poor cognitive abilities are more susceptible to stressful stimuli; a hypothesis that has been supported by other researchers as well (19). That is, employees with greater executive functions probably are less intimidated by the stressful working conditions (e.g., high job demands), they are more capable in adjusting their behavior when they face new or challenging work-related tasks and as a result, they experience greater personal efficacy; resulting in a positive spillover effect (87). An observation which could also explain the cognitive resilience among non-clinical burnout populations.

Present results extend our baseline findings (32) by indicating that high scores on certain burnout dimensions can be long-lasting and emphasize the complexity of the nature of relationship between burnout and cognitive functions. Current findings could explain the results of Castaneda et al. (31) who observed positive associations between burnout and cognitive performance, but the cross-sectional design of their study did not allow for the examination of reciprocal associations. Hence, it is possible that participants’ high executive functions led to better cognitive performance. Our findings are in contrast with previous longitudinal studies which suggested that executive functioning deficits are a burnout consequence (22, 23). However, Lemonaki et al. (23) indicated that poorer cognitive flexibility is associated with a later burnout onset. Moreover, our results are also in disagreement with cross-sectional studies that did not observe any significant links between burnout and cognitive functioning (30, 88) as well as with the cross-sectional study of van Dijk et al. (29) who found that non-clinical burnout employees exhibited lower performance comparing to healthy individuals on demanding working memory tasks.

An additional remark concerns the arguments of previous studies regarding the inclusion of cognitive impairments as a core burnout dimension (5, 6). Current findings give partial support to the burnout conceptualization which considers cognitive impairments as a core burnout dimension. Although, visuospatial skills were identified as a burnout consequence, present results show that the relationship between burnout and cognitive functioning is more complex than previously might have been thought. Future studies need not only to investigate the dynamic relationship between burnout and cognitive functions but also need to explore a wide range of cognitive domains in order to gain a more complete picture of this relationship before we consider cognitive impairments as a burnout dimension.

### The relationship of depression, anxiety and perceived family support with cognitive functioning

Present results did not show any effects of self-reported depression and anxiety on cognitive functioning; a result that comes in contrast with studies that observed cognitive impairments in individuals suffering from depression (89) and anxiety (90). Most studies identifying cognitive deficits in depression and anxiety examined clinical populations. In the present study the participants reported mostly mild depression and anxiety feelings. Thus, it is possible that cognitive impairment is mainly observed among clinical populations either as a consequence or a cause of depression and/or anxiety. Indeed, it is possible that individuals who experience cognitive deficits might manifest depressive and/or anxiety symptoms due to the realization of those deficits (91). Importantly, as depression and anxiety patients often receive medication treatments, one cannot rule out the possibility that the cognitive deficits could emerge as a result of the medication the patients receive and not from their clinical condition *per se*.

Concerning perceived family support, it had a positive impact on long-term visuospatial memory. Other studies have also shown the beneficial effects levels of family (59) and social (92) support on cognitive performance. It has been proposed that perceived support and social engagement challenge our cognitive system by stimulating it through the interaction with the members of our social and family networks (93) and thus, enhances individuals’ cognitive reserve (33). Hence, this cognitive stimulation through our social interactions could result to better cognitive performance. The importance of social support becomes evident from research evidence which suggests that psychosocial factors can influence the cognitive functioning of individuals who are at-risk in developing dementia (94).

### Burnout, depression, anxiety and perceived family support

Present results revealed reciprocal associations between burnout and depression screening, and burnout and anxiety displaying significant and positive longitudinal stability. These results are in agreement with previous longitudinal studies where a reciprocal relationship between burnout and depression was observed (43). Importantly, all three burnout components were significantly associated with both depression screening and anxiety suggesting that the two latter psychological phenomena are related equally to overall burnout levels and not to specific burnout components; results that indicate the importance of research studies taking into consideration the multifactorial dimension of burnout and not only its certain components. However, the effect sizes among the variables when the three burnout components were set as the predictors were very strong while the effect sizes when depression screening and anxiety predicted burnout were weaker. This observation advocates toward the hypothesis that burnout could be a precursor of these two psychological phenomena, giving support to the approach of Tavella et al. (39) who argued that depressive symptoms might arise as a consequence of burnout’s effects. Indeed, burned-out employees might be more susceptible in developing depression and anxiety. Hakanen and Schaufeli (41) in their three-wave study showed that burnout predicts depression and not the opposite. However, considering the fact that most longitudinal studies support reciprocal paths, this view cannot be considered strong enough to support the hypothesis that burnout is an antecedent of depression and anxiety. More longitudinal studies examining the reciprocal associations of burnout with depression and anxiety are needed in order to gain a better understanding of how these associations develop over time. Moreover, the investigation of the role of the context (i.e., work-specific and context-general) and how it affects individuals’ mental health could provide further insights on the nature of the two relationships (41).

An additional goal of this study was to examine the bidirectional influences of perceived family support with burnout, depression screening and anxiety. Our results showed robust evidence that perceived family support has reciprocal associations with certain aspects of burnout, depression screening and anxiety in the directions expected. Specifically, high levels of perceived family support were found to decrease cynicism, depression screening and anxiety over time whereas high cynicism, depression screening and anxiety levels negatively affected perceived family support. Nevertheless, the effects of perceived family support on these three factors were higher comparing to the opposite path. These results emphasize the importance of family context in mental health as it can either protect against the development of psychological problems or it can be disrupted by pre-existing psychological difficulties. This observation could be explained on the basis of the conservation of resources model (COR) (95). COR model’s key tenet is that individuals are prompted to protect their resources (i.e., everything they value such as energy, relationships etc.) and when these resources are either threatened or lost, then stress emerges. The COR model indicates the importance of social support which acts as a resource pool when the personal resources have been exhausted (96). Present results recognize the role of perceived family support as a potential protective mechanism against mental health problems. That is, distressed individuals might turn to their family members in order to help them compensate for their lost resources. The COR model could also explain this reversed effect. When negative feelings start to emerge, then individuals start to avoid interactions with their family members as an attempt to preserve their energy resources; as quality family relationships involve active participation among family members, posing demands on the distressed individual’s already limited resources. The reversed associations also give support to Coyne’s interactional theory of depression (1976) and indicate that, not only depression, but other mental health difficulties as well can result in negative interactions with family members and poorer quality of family relations.

Surprisingly, perceived family support was significantly associated only with cynicism, but not with the exhaustion and personal efficacy burnout dimensions. The main conceptual burnout model posits that the three burnout dimensions develop in a subsequent order. That is, high exhaustion feelings lead to high cynicism levels which, consequently, leads to inefficacy (97). However, others have considered cynicism as a coping mechanism against stress and burnout (98, 99). It has been proposed that cynicism can result in negative associations with prosocial behavior and interpersonal conflicts (100, 101). Prosocial behavior is a social behavior that enhances both the wellbeing and integrity of other people or society as a whole *via* committing in positive behaviors such as helping, cooperating and supporting (102). Hence, a possible interpretation is that high levels of cynicism could result in a more cynical behavior toward family members.

### Practical implications

The results indicate that visuospatial functioning deficits are a non-clinical burnout consequence. Visuospatial functions are responsible for the mental representation of objects and spatial relationships, and they allow us to navigate through our environment (103). Thus, these observations posit significant concerns regarding employees’ personal (e.g., driving) and work life, especially for those occupations where integral visuospatial abilities are of importance such as professional drivers, engineers and surgeons as diminished visuospatial skills could put at risk client and patient safety [for a mini review see Koutsimani and Montgomery (104)]. Executive functions are also of importance when encountering stressful situations in the workplace while well-established skills can facilitate performance at the initial burnout stages. As prevention is more desirable than intervention, the building of prevention strategies focusing on the enhancement of executive functions as well as employees’ cognitive strengths could help tackle those conditions resulting in burnout. Although we observed significant links between burnout and cognitive functions, our results are not robust enough to suggest that cognitive impairments could be a core burnout dimension. Moreover, present results support the notion that burnout is distinct from both depression and anxiety. However, these three mental health problems have reciprocal reinforcing associations. Practitioners could build intervention programs focusing on the alleviation of the symptoms of all three psychological phenomena, as all three appear to spill over to individuals’ work and personal life areas. Based on the reinforcing associations, intervention programs could focus on diminishing depression and anxiety feelings by helping employees to gain a more positive perspective toward life which could lead toward a more satisfied outlook of their work, and vice versa. Considering the mutual relationships between perceived family support, cynicism, depression and anxiety, practitioners should consider in adapting techniques focusing on the enhancement of one’s family ties; referring individuals with mental health problems to family therapy could be beneficial for both patients and their families. Finally, practitioners could also consider integrating the strengthening of family ties within cognitive training and neurorehabilitation programs.

Overall, the results of the present study offer important insights on the relationship of burnout with cognitive functioning, depression screening and anxiety and they also underline the important role of family context. Future longitudinal studies are needed in order to disentangle the role of these factors.

### Limitations

The present study is not without limitations. The sample size was relatively small, thus making our results more susceptible in Type I error. Future studies with larger sample sizes will allow a better comprehension of the variables’ predictive associations. Considering that we used the snowball sampling method we cannot rule out the possibility of a potential sampling bias and a higher margin of error. Although no statistically significant differences were observed between the participants who continued with the study and the participants who dropped out, there was a 50% dropout rate at T3 which could result in a bias dropout and thus, to a loss of representativeness of the sample. Indeed, participants at T3 reported higher cynicism levels hence, suggesting that participants’ non-response occurred not at random (NMAR) (105). Moreover, burnout, depression, anxiety and perceived family support were examined through self-reported questionnaires, thus participants’ answers could be affected by potential self-report biases. In addition to this, both depression and anxiety were assessed *via* a single scale; i.e., the HADS, Although HADS has been found to be appropriate for non-clinical populations (67), the scale covers a limited array of the symptoms characterizing both depression and anxiety. Scales that cover the full range of depression and anxiety symptoms could have provided different results. Even though we measured all studied variables across three different time points, we cannot infer causality. Due to practical reasons, we chose to assess the variables of interest in an 8 and 17-month period interval after the baseline examination. However, the chosen time lags between each wave might not have been sufficient to detect real changes. Indeed, the lack of consensus on the appropriate time lags and thus, the arbitrary time points that are chosen is in itself a methodological limitation in longitudinal studies (106); longer, or shorter, time frames between time intervals in future longitudinal studies could provide further information on the exact associations among these relationships. Lastly, although we examined a broad range of cognitive functions of varying difficulty, more complex cognitive tasks when examining non-clinical burnout employees in future studies should be considered. In fact, although our results show that visuospatial deficits are a cynicism outcome, we cannot overlook the possibility of other cognitive functions underlying this relationship. That is, we assessed visuospatial skills with the administration of a constructional task (i.e., TCFT-copy condition); a task that integrates visual perception with motor response and thus, it includes a spatial component (107). Performance on constructional tasks can be affected by other functions including basic attentional abilities (i.e., sustained and visual attention, alertness and focus). Motor skill performance has been associated with attention (visual and alertness) (108). Although we measured for executive attention (*via* the Stroop task and TMT—part B) and visual attention (*via* TMT part—A), it is possible that deficits in these areas might have been missed due to the lack of complexity of the tasks on the general population. The TCFT—copy condition demands competent attention and focus in order to recognize the complex figure and approach the visual information (109). Thus, basic attention deficits that might went undetected could have mediated the relationship between cynicism and visuospatial skills; emphasizing the need for thorough cognitive screening when examining healthy populations.

## Conclusion

As non-clinical burnout refers to the early burnout stages where employees are still working, it is of importance to detect its symptoms at this early phase in order to prevent individuals from reaching clinical stages. This study emphasizes the role of executive functions in employees’ mental (emotional and cognitive) resilience while they also reveal the adverse effects of non-clinical burnout in visuospatial abilities; a cognitive domain that has not received much attention in the relevant literature. Although moderate burnout levels (i.e., high cynicism levels) are sufficient to affect optimal cognitive performance, high executive function abilities can protect against both cognitive decline and certain burnout aspects. Our evidence points to the distinction of burnout from depression and anxiety but also support that burnout is not independent from the two psychological syndromes. Additionally, the findings of this study highlight the role of other psychosocial non-work-related factors in both mental health and cognition. Overall, present findings reveal the practical implications of the early detection of burnout and the role of perceived support in both cognitive functioning and mental health. It is of importance to know which factors can affect cognitive performance and mental health, as this will help in the development of more targeted intervention programs.

## Data availability statement

The raw data supporting the conclusions of this article will be made available by the authors, without undue reservation.

## Ethics statement

Ethical review and approval was not required for the study on human participants in accordance with the local legislation and institutional requirements. The patients/participants provided their written informed consent to participate in this study.

## Author contributions

PK and AM: conceptualization, methodology, resources, software, and visualization. PK: data curation, formal analysis, funding acquisition, investigation, and writing—original draft. AM: project administration, supervision, validation, and writing—review and editing. Both authors have read and agreed to the published version of the manuscript.
