# Supplementary material for: Cognitive functioning in non-clinical burnout: Using cognitive tasks to disentangle the relationship in a three-wave longitudinal study
Source: Front Psychiatry. 2022 Aug 17;13:978566. doi: 10.3389/fpsyt.2022.978566 (PMC9428402; doi:10.3389/fpsyt.2022.978566)
Supplement: Supplementary file 1 [file Data_Sheet_1.docx]

***Supplementary Material***

**Supplementary Table 1**. Summary of test – retest reliability scores for cognitive tasks across the three time points.

| Cognitive Task | Time 1 Time 2 Time 3  *M SD M SD M SD* | | | Cronbach’s α | Intraclass  Correlation | 95% Confidence Interval  Lower Limit Upper Limit | |
| --- | --- | --- | --- | --- | --- | --- | --- |
| Taylor Complex Figure Test |  |  |  | 0.77 | 0.77 | 0.72 | 0.82 |
| Copy | 33.57 2.74 | 32.70 2.60 | 32.36 2.81 | 0.78 | 0.77 | 0.64 | 0.86 |
| Immediate Recall | 25.31 5.22 | 26.52 5.16 | 27.58 5.21 | 0.89 | 0.88 | 0.80 | 0.93 |
| Delayed Recall | 24.77 5.04 | 25.86 5.30 | 27.45 4.79 | 0.86 | 0.84 | 0.74 | 0.91 |
| Recognition | 19.27 2.15 | 19.91 1.97 | 20.70 1.89 | 0.54 | 0.51 | 0.24 | 0.69 |
| Short Story Recall |  |  |  | 0.75 | 0.75 | 0.67 | 0.80 |
| Immediate Recall | 21.43 5.45 | 22.48 4.44 | 23.34 4.01 | 0.78 | 0.78 | 0.65 | 0.86 |
| Delayed Recall | 11.69 2.92 | 12.01 2.08 | 12.40 2.16 | 0.74 | 0.75 | 0.61 | 0.84 |
| Prospective Memory | 8.88 3.93 | 8.38 3.73 | 7.63 3.89 | 0.68 | 0.66 | 0.48 | 0.79 |
| Stroop Test |  |  |  | 0.71 | 0.71 | 0.64 | 0.77 |
| Word | 104.08 15.49 | 109.33 11.79 | 111.41 12.76 | 0.72 | 0.71 | 0.55 | 0.82 |
| Congruent | 74.24 10.23 | 77.95 11.95 | 78.50 10.77 | 0.83 | 0.82 | 0.71 | 0.89 |
| Incongruent | 47.64 9.81 | 50.29 9.32 | 52.30 8.74 | 0.81 | 0.80 | 0.69 | 0.87 |
| Trail Making Test |  |  |  | 0.61 | 0.61 | 0.50 | 0.70 |
| Part A | 38.68 17.30 | 30.84 11.73 | 27.56 11.80 | 0.86 | 0.82 | 0.66 | 0.90 |
| Part B | 72.02 27.71 | 62.30 25.72 | 57.14 22.19 | 0.79 | 0.77 | 0.63 | 0.86 |
| Digit Span |  |  |  | 0.70 | 0.70 | 0.66 | 0.78 |
| Forward | 9.27 2.08 | 9.93 2.15 | 9.85 2.22 | 0.83 | 0.82 | 0.73 | 0.89 |
| Backwards | 9.22 2.48 | 9.55 2.06 | 9.30 2.37 | 0.86 | 0.86 | 0.78 | 0.91 |
| Ascending | 9.86 2.50 | 10.11 2.21 | 10.00 2.19 | 0.73 | 0.73 | 0.58 | 0.83 |
| Corsi Span (backwards) | 28.43 5.52 | 29.68 4.78 | 28.58 4.71 | 0.80 | 0.80 | 0.69 | 0.87 |

**Supplementary Table 2**. Correlation coefficients between burnout, depression, anxiety, family support and cognitive tasks at T1 (*N*=104).

| Variables | TCFT-C | TCFT-IR | TCFT-DR | TCFT Rec. | Short Story-IR | Short Story-DR | Prosp.Mem. | STROOP-W | STROOP-C | STROOP-CW | STROOP-Int. | Digit Span-FW | Digit Span-Back | Digit Span-Asc | Digit Span-tot. | Corsi Span- back | TMT-A | TMT-B |
| --- | --- | --- | --- | --- | --- | --- | --- | --- | --- | --- | --- | --- | --- | --- | --- | --- | --- | --- |
| 1.E | .02 | .02 | -.05 | -.07 | .00 | .10 | .02 | -.02 | -.03 | -.02 | -.00 | .00 | .05 | -.01 | -.08 | -.02 | .03 | .01 |
| 2.CY | -.19* | -.16 | -.07 | -.09 | .04 | -.01 | .02 | .19* | .02 | -.03 | -.10 | -.07 | -.04 | -.09 | -.09 | -.06 | .04 | .16 |
| 3.PE | .17 | -.03 | .04 | .00 | .06 | .10 | .10 | .00 | .07 | .20* | .19 | -.00 | .07 | .01 | .02 | .01 | .04 | .00 |
| 4.HADS-D | -.10 | -.13 | -.08 | -.07 | -.04 | -.00 | -.07 | .08 | -.04 | -.01 | .01 | .00 | -.00 | -.06 | -.03 | -.00 | .05 | .09 |
| 5. HADS-A | -.07 | -.14 | -.12 | -.07 | .04 | .13 | -.00 | .06 | -.01 | -.07 | -.10 | .08 | .04 | -.04 | -.00 | .00 | .07 | .03 |
| 6. FSS | .01 | .21 | .32** | .07 | .07 | .14 | -.06 | -.08 | .09 | .08 | .05 | .24* | -.00 | .19 | .04 | .11 | -.14 | -.20 |

*Note*. E= Exhaustion, CY= Cynicism, PE = Personal Efficacy, HADS-D = Hospital Anxiety and Depression Scale-Depression, HADS-A = Hospital Anxiety and Depression Scale-Anxiety, FSS = Family Support Scale TCFT-C = Taylor Complex Figure Test-Copy trial, TCFT-IR = Taylor Complex Figure Test-Immediate Recall trial, TCFT-DR = Taylor Complex Figure Test-Delayed Recall trial, TCFT-Rec. = Taylor Complex Figure Test-Recognition trial, Short Story-IM= Short Story Immediate Recall trial, Short Story-DR= Short Story Delayed Recall trial, Prosp.Mem.. = Prospective Memory, STROOP- W = STROOP Word, STROOP-C = STROOP Color, STROOP-CW = STROOP Color-Word, STROOP-Int. = STROOP Interference, Digit Span-FW = Digit Span Forward, Digit Span-Back. = Digit Span Backwards, Digit Span-Asc. = Digit Span Ascending Order, Digit Span- tot. = Digit Span total score, Corsi Span back. = Corsi Span backwards, TMT-A = Trail Making Test, Part A, TMT-B = Trail Making Test, Part B.

**. *p* < 0.01 level (2-tailed). *. *p* < 0.05 level (2-tailed).

| Variables | TCFT-C | TCFT-IR | TCFT-DR | TCFT Rec. | Short Story-IR | Short Story-DR | Prosp.Mem. | STROOP-W | STROOP-C | STROOP-CW | STROOP-Int. | Digit Span-FW | Digit Span-Back | Digit Span-Asc | Digit Span-tot. | Corsi Span- back | TMT-A | TMT-B |
| --- | --- | --- | --- | --- | --- | --- | --- | --- | --- | --- | --- | --- | --- | --- | --- | --- | --- | --- |
| 1.E | -.21 | -.10 | -.06 | -.17 | -.04 | .12 | -.09 | .03 | -.07 | .11 | .24* | -.05 | -.15 | -.02 | -.12 | -.09 | -.07 | .07 |
| 2.CY | -.07 | -.13 | -.12 | -.00 | -.06 | .05 | -.08 | .10 | -.03 | .02 | .06 | -.07 | -.13 | -.22 | -.17 | -.04 | .01 | .06 |
| 3.PE | .08 | .03 | .02 | -.09 | -.05 | -.01 | .16 | .20 | .13 | .09 | -.05 | .04 | -.02 | .16 | .12 | -.02 | .08 | -.08 |
| 4.HADS-D | .08 | -.15 | -.06 | .08 | -.01 | .02 | .10 | .09 | -.19 | -.02 | .03 | -.15 | -.20 | -.08 | -.25* | -.15 | .10 | .06 |
| 5. HADS-A | .05 | -.21 | -.15 | .07 | -.10 | .02 | .16 | .14 | -.13 | -.00 | .02 | -.15 | -.06 | .06 | -.12 | -.16 | .10 | .13 |
| 6. FSS | .22 | .39** | .38** | .40** | .15 | .07 | .01 | -.01 | .20 | .04 | .00 | .07 | .20 | .23 | .30* | .31* | -.23 | -.48** |

**Supplementary Table 3**. Correlation coefficients between burnout, depression, anxiety, family support and cognitive tasks at T2 (*N*=72).

*Note*. E= Exhaustion, CY= Cynicism, PE = Personal Efficacy, HADS-D = Hospital Anxiety and Depression Scale-Depression, HADS-A = Hospital Anxiety and Depression Scale-Anxiety, FSS = Family Support Scale TCFT-C = Taylor Complex Figure Test-Copy trial, TCFT-IR = Taylor Complex Figure Test-Immediate Recall trial, TCFT-DR = Taylor Complex Figure Test-Delayed Recall trial, TCFT-Rec. = Taylor Complex Figure Test-Recognition trial, Short Story-IM= Short Story Immediate Recall trial, Short Story-DR= Short Story Delayed Recall trial, Prosp.Mem.. = Prospective Memory, STROOP- W = STROOP Word, STROOP-C = STROOP Color, STROOP-CW = STROOP Color-Word, STROOP-Int. = STROOP Interference, Digit Span-FW = Digit Span Forward, Digit Span-Back. = Digit Span Backwards, Digit Span-Asc. = Digit Span Ascending Order, Digit Span- tot. = Digit Span total score, Corsi Span back. = Corsi Span backwards, TMT-A = Trail Making Test, Part A, TMT-B = Trail Making Test, Part B.

**. *p* < 0.01 level (2-tailed). *. *p* < 0.05 level (2-tailed).

**Supplementary Table 4**. Correlation coefficients between burnout, depression, anxiety, family support and cognitive tasks at T3 (*N*=55).

| Variables | TCFT-C | TCFT-IR | TCFT-DR | TCFT Rec. | Short Story-IR | Short Story-DR | Prosp.Mem. | STROOP-W | STROOP-C | STROOP-CW | STROOP-Int. | Digit Span-FW | Digit Span-Back | Digit Span-Asc | Digit Span-tot. | Corsi Span- back | TMT-A | TMT-B |
| --- | --- | --- | --- | --- | --- | --- | --- | --- | --- | --- | --- | --- | --- | --- | --- | --- | --- | --- |
| 1.E | -.01 | -.18 | -.09 | .02 | -.13 | -.13 | .06 | -.09 | .00 | .13 | .17 | -.20 | -.15 | -.17 | -.21 | -.06 | .04 | .04 |
| 2.CY | .03 | .04 | -.03 | .01 | -.00 | .03 | .02 | -.03 | .07 | .24 | .30* | -.13 | .04 | -.17 | -.21 | .05 | -.08 | -.08 |
| 3.PE | .25 | -.04 | .04 | -.03 | -.02 | -.10 | -.11 | -.01 | -.02 | .14 | .19 | .05 | .06 | .20 | .13 | -.02 | -.13 | -.13 |
| 4.HADS-D | .12 | .00 | .04 | -.01 | .07 | .14 | .08 | -.12 | -.06 | .18 | .03 | .04 | .07 | -.08 | .14 | .06 | -.02 | .02 |
| 5. HADS-A | -.01 | -.03 | .03 | -.09 | .08 | .20 | .12 | -.20 | -.13 | .08 | .21 | -.00 | .02 | -.19 | -.07 | -.04 | .12 | .14 |
| 6. FSS | -.09 | -.00 | -.09 | .32* | .03 | -.06 | .10 | .10 | .15 | -.05 | -.13 | .27 | .27 | .34* | .33* | .15 | -.13 | -.33* |

*Note*. E= Exhaustion, CY= Cynicism, PE = Personal Efficacy, HADS-D = Hospital Anxiety and Depression Scale-Depression, HADS-A = Hospital Anxiety and Depression Scale-Anxiety, FSS = Family Support Scale TCFT-C = Taylor Complex Figure Test-Copy trial, TCFT-IR = Taylor Complex Figure Test-Immediate Recall trial, TCFT-DR = Taylor Complex Figure Test-Delayed Recall trial, TCFT-Rec. = Taylor Complex Figure Test-Recognition trial, Short Story-IM= Short Story Immediate Recall trial, Short Story-DR= Short Story Delayed Recall trial, Prosp.Mem.. = Prospective Memory, STROOP- W = STROOP Word, STROOP-C = STROOP Color, STROOP-CW = STROOP Color-Word, STROOP-Int. = STROOP Interference, Digit Span-FW = Digit Span Forward, Digit Span-Back. = Digit Span Backwards, Digit Span-Asc. = Digit Span Ascending Order, Digit Span- tot. = Digit Span total score, Corsi Span back. = Corsi Span backwards, TMT-A = Trail Making Test, Part A, TMT-B = Trail Making Test, Part B.

*. *p* < 0.05 level (2-tailed).
